# Nuclear DNA Content Variation in Life History Phases of the Bonnemasoniaceae (Rhodophyta)

**DOI:** 10.1371/journal.pone.0086006

**Published:** 2014-01-22

**Authors:** Noemi Salvador Soler, Amelia Gómez Garreta, Mª Antonia Ribera Siguan, Donald F. Kapraun

**Affiliations:** 1 Education Department, Universidad Autónoma de Chile, Temuco, Chile; 2 Laboratori de Botànica, Facultat de Farmàcia, Universitat de Barcelona, Barcelona, Spain; 3 Department of Biology & Marine Biology, University of North Carolina Wilmington, Wilmington, North Carolina, United States of America; University of Connecticut, United States of America

## Abstract

Nuclear DNA content in gametophytes and sporophytes or the prostrate phases of the following species of Bonnemaisoniaceae (*Asparagopsis armata, Asparagopsis taxiformis, Bonnemaisonia asparagoides, Bonnemaisonia clavata* and *Bonnemaisonia hamifera*) were estimated by image analysis and static microspectrophotometry using the DNA-localizing fluorochrome DAPI (4′, 6-diamidino-2-phenylindole, dilactate) and the chicken erythrocytes standard. These estimates expand on the Kew database of DNA nuclear content. DNA content values for 1C nuclei in the gametophytes (spermatia and vegetative cells) range from 0.5 pg to 0.8 pg, and for 2C nuclei in the sporophytes or the prostrate phases range from 1.15–1.7 pg. Although only the 2C and 4C values were observed in the sporophyte or the prostrate phase, in the vegetative cells of the gametophyte the values oscillated from 1C to 4C, showing the possible start of endopolyploidy. The results confirm the alternation of nuclear phases in these Bonnemaisoniaceae species, in those that have tetrasporogenesis, as well as those that have somatic meiosis. The availability of a consensus phylogenetic tree for Bonnemaisoniaceae has opened the way to determine evolutionary trends in DNA contents. Both the estimated genome sizes and the published chromosome numbers for Bonnemaisoniaceae suggest a narrow range of values consistent with the conservation of an ancestral genome.

## Introduction

The marine red algal genera *Asparagopsis* and *Bonnemaisonia* (Bonnemaisoniaceae, Bonnemaisoniales) have been the subject of numerous life history studies [1], [2], [3], [4], [5], [6], invasive species ecology [7], [8], [9], phylogeography [10], [11], [12] and potential applications of their bioactive metabolites [13], [14]. Despite continuing interest in members of this order, modern molecular techniques are only now beginning to overcome a history of pervasive taxonomic and nomenclatural confusion [8], [15], [6]. Although the Bonnemaisoniales was separated from the Nemaliales on the basis of their then known alternation of generations [1], it is now understood that this life history pattern lacks taxonomic significance and many orders of red algae are heterogeneous with regard to life history [16]. The distinction of these two orders is now generally recognized on the basis of sexual reproduction and cystocarp development [17], ultrastructural details of pit plugs and plastids [18], [19] as well as molecular studies [20], [21].

The Bonnemaisoniales, as originally proposed [1], is characterized by a heteromorphic life history. The *Asparagopsis* genus has a much branched erect gametophyte, and a tufted sporophyte (“*Falkenbergia*” stage) with polysiphonous axes. The *Bonnemaisonia* genus also has a much branched erect gametophyte, and a mycroscopic and postrate sporophyte, (“*Hymenoclonium*” stage), or filamentous and tufted (“*Trailliella*” stage). According to Dixon [22], the information available for members of these taxa indicates both a ‘Bonnemaisonia’-type life history as well as a direct development of gametophytes from vegetative branches of the assumed diploid sporophyte with an absence of tetrasporogenesis [23], [24], [25], [26]. In addition, in *Bonnemaisonia asparagoides* (Woodward) C. Agardh and *Bonnemaisonia clavata* Hamel somatic meiosis has been described [4], [6] as reported in the ‘Lemanea’-type life history [27]. Despite the numerous studies carried out on the life history of the Bonnemaisoniales, the sequence of nuclear phases has been demonstrated only in *B*. *asparagoides* and *B*. *clavata* [6].

Microspectrophotometry with the DNA-localizing fluorochrome DAPI (4′, 6-diamidino-2-phenylindole, dilactate) was used successfully to demonstrate an alternation of ploidy levels associated with meiosis and sexual reproduction in red algae [28], [29]. Among these algae there are members of Batrachospermales and Thoreales which have a ‘Lemanea’-type life history [30], [27], such us some *Bonnemaisonia* species.

The present research of nuclear DNA contents in Bonnemaisoniaceae was initiated to determine the extent of nuclear DNA content variation, to identify any correlation between genome size and phylogeny, and to corroborate an alternation of haploid and diploid nuclear DNA contents in gametophyte and sporophyte/prostrate phases, respectively.

## Materials and Methods

### Source of specimens

“The locations for plant collections in this study were not privately-owned or protected in any way, so no specific permissions were required for these locations/activities; also none of the species used in this study involve endangered or protected species”.

Five species of Bonnemaisoniaceae were collected from the Mediterranean [Aiguafreda and Llançà (Girona), Porto Colom (Majorca)] and Atlantic [Cabo Cruz (A Coruña), Zumaya (Guipúzcoa)] coasts of Spain: *Bonnemaisonia hamifera* Hariot (including *Trailliella intricata* Batters), *B*. *asparagoides*, *B*. *clavata*, *Asparagopsis armata* Harvey (including *Falkenbergia rufolanosa* (Harvey) F. Schmitz) and *Asparagopsis taxiformis* (Delile) Trevisan (including *Falkenbergia hillebrandii* (Bornet) Falkenberg) (Table 1). Due to the difficulty in obtaining ‘Hymenoclonium’ phases of *B*. *clavata* and *B*. *asparagoides*, these phases were cultured in the laboratory from carpospores which produced gametophytes [15].

**Table 1:** Nuclear DNA content of Bonnemaisoniales. Data standardized to the DNA level of chicken erythrocytes (RBC = 2.4 pg).

						Nuclear Genome Size (pg)			
Species	Location	Date	Phase	Cell Type	N° of Nuclei examined	1C	2C	4C	Method
*Asparagopsis armata*	Llançà	04/02/2007	G	C	11			3.2±0.6	IA
	″	″	″	V	137		1.7±0.2		IA
	″	″	″	Sp	51		1.8±0.3		IA
	″	″	″	Sp	123	0,65±0.1			M
	″	″	S	V	157		1.6±0.1		M
	″	″	S	V	14			2.7±0.4	M
*Asparagopsis taxiformis*	Porto Colom	06/05/2007	G	V	63		1.8±0.2		IA
	″	″	G	V	17			2,4±0.4	IA
	″	″	G	V	10			2.9±0.4	M
	″	″	G	Sp	160	0.7±0.2			M
	″	″	G	Sp	29	0.65±0.1			IA
	″	″	G	Sp	67		1.7±0.1		IA
	″	″	G	Sp	4		1.6±0.1		M
	″	″	S	V	102		1.7±0.3		M
*Bonnemaisonia asparagoides*	Aiguafreda	30/05/2007	G	V	51	0.6±0.1			M
	″	″	G	V	46	0.6±0.2			IA
	″	″	G	Sp	21	0.5±0.1			M
	″	″	G	Sp	12	0.6±0.2			IA
	″	″	G	V	30		1.1±0.2		M
	″	″	G	V	34		1.45±0.2		IA
	″	″	G	C	5			2.3±0.2	IA
	culture		G	V	29			2.2±0.1	IA
	culture		G	C	99		1.65±0.2		IA
	culture		S	V	168		1.15±0.2		M
	culture		S	V	183			2.25±0.1	IA
*Bonnemaisonia clavata*	Cabo Cruz	13/06/2006	G	Sp	51	0.7±0.1			M
	″	″	G	Sp	13		1.1±0.1		M
	Aiguafreda	30/05/2007	G	V	8	0.6±0.2			IA
	″	″	G	V	25		1.6±0.1		IA
	″	″	G	Sp	83	0.6±0.2			IA
	″	″	G	C	71			2.2±0.2	IA
	culture		G	C	84		1.6±0.1		IA
	culture		S	V	87		1.2±0.2		M
	culture		S	V	183			2.2±0.2	IA
*Bonnemaisonia hamifera*	Zumaya	07/10/2006	G	V	104	0.8±0.2			M
	″	″	G	V	89		1.5±0.3		M
	″	″	G	V	16			2.7±0.7	M
	″	″	S	V	172		1.35±0.2		IA
	″	″	S	V	110			3.2±0.6	M
*^1^Delisea plumosa*	New Zealand		G	V	47		1.0±0.2		M
*^1^Ptilonia willana*	New Zealand		G	V	165	0.6±0.1			M

Unpublished data from Kapraun.

G  =  gametophe, S  =  sporophytic/prostrate phases, C  =  carpospora, V  =  vegetative cell, Sp  =  spermatia, IA  =  image analysis, M  =  microspectrophotometry.

### Nuclear DNA content estimates

Algal specimens were fixed in Carnoy's solution (3∶1 95% ethanol: glacial acetic acid) and stored in 70% ethanol at 4°C [28]. Preserved material was rehydrated in water and softened in 5% w/v EDTA for 12 h [31]. Algal material was squashed, transferred to cover slips treated with subbing solution, air dried and stained with DAPI (0.5 µg mL^-1^) (Sigma Chemical Co., St. Louis, MO 63178) as previously described [31], [32]. Nuclear DNA content estimates based on microspectrophotometry with DAPI followed procedures specified previously [31], [32] using a protocol modified after Goff & Coleman [31]. This method was carried out at the University of North Carolina Wilmington. Nuclear DNA content estimates based on image analysis of DAPI-stained specimens followed a procedure modified from Kapraun & Dunwoody [33] and Choi *et al*. [34] using a Cooled CCD Miramax RTE 782-Y high performance digital camera placed on a Leica DMRB fluorescence microscope and subsequently analyzed using MetaMorph software (Molecular Devices, Toronto, Canada). This method was carried out at the University of Barcelona. Fluorescence intensity (I_f_) values were obtained from image analysis and microspectrophotometry for algal specimens [35], [33].

DAPI binds by a non-intercalative mechanism to adenine and thymine rich regions of DNA which contain at least four A-T base pairs [36]. Chicken erythrocytes (RBC) with a DNA content of 2.4 pg [37] were used as standard to quantify nuclear DNA contents. RBC can be used directly as a standard for determining amounts of DNA only when the A-T contents of both standard and experimental DNA are equivalent [38]. Chicken has a nuclear DNA base composition of 42–43 mol % G + C [39]. Published data indicate similar mean mol% values for the Rhodophyta [40], [41], [42], [43], [44]. Algae investigated in this study are assumed to have a similar range of base pair compositions, and linearity is presumed between DAPI-DNA binding in both RBC and algal samples [43]. Nuclear DNA contents were estimated by comparing the I_f_ (intensity of fluorescence) values of the RBC standard and algal samples [32]. Nuclear DNA content data for these and other red algae are incorporated into the database of plant genome sizes [28], [45] compiled and hosted by the Royal Botanic Gardens (RBG) Kew web page (http://www.rbgKew.org.uk/cval/homepage.html).

### Assignment of ploidy level

Assignment of estimated nuclear DNA contents to specific C-values is presumptive in that no karyological research was conducted on the algal samples used for nuclear DNA content estimates. Nuclear DNA contents, referred to as C-values [46], represent multiples of the minimum amount of DNA corresponding to the non-replicated haploid chromosome complement [47], [48]. In the present study, the numerical relationship between the C-values and the I_f_ values obtained was established from the spermatia which can have a haploid genome = 1C (G_1_) or a replicated haploid genome = 2C (G_2_), according to Goff & Coleman [31].

## Results

Nuclear genome size estimates (pg±SD) were obtained for five species of Bonnemaisoniaceae. Table 1 includes information about location, sampling dates, cycle phases and cell types studied, and the number of nuclei examined, as well as all the results obtained. Table 2 summarizes the mean estimates for 1C and 2C values.

**Table 2:** Nuclear DNA means content of Bonnemaisoniales. 1C values (spermatia) and 2C values (sporophytic/prostrate phases).

		Nuclear Genome Size (pg)	
Species	Reproductive Phase	1C	2C
*Asparagopsis armata*	G	0.7±0.1	
(* = *‘Falkenbergia’ phase)	S		1.6±0.1
*Asparagopsis taxiformis*	G	0.7±0.2	
(* = *‘Falkenbergia’ phase)	S		1.7±0.3
*Bonnemaisonia hamifera*	G		
(* = *‘Trailliella’ phase)	S		1.4±0.2
*Bonnemaisonia asparagoides*	G	0.6±0.2	
( = ‘Hymenoclonium’ phase)	S		1.2±0.2
*Bonnemaisonia clavata*	G	0.7±0.3	
( = ‘Hymenoclonium’ phase)	S		1.2±0.2

G = gametophyte, S = sporophytic/prostrate phase.

The members of the Bonneamaisoniaceae studied showed gametophytes with DNA content values from 1C (0.5–0.8 pg) to 4C (2.2–2.9 pg), and sporophytes or prostrate phases with values from 2C (1.15–1.7 pg) to 4C (2.2–3.2 pg). A similar range of 1C (0.55–0.85 pg) was obtained by extrapolation from the 2C mean values found in the sporophyte or the prostrate phase (Table 1). For all the species studied, the results obtained by microspectrophotometry and by image analysis were similar.

Concerning reproductive structures, the DNA nuclear contents of spermatia and carpospores were analyzed. The spermatia values were obtained for the two species of *Asparagopsis* (1C = 0.7 pg), for *B. asparagoides* (1C = 0.6 pg) and for *B. clavata* (1C = 0.7 pg). The carpospores values were also obtained for of *A. armata* (4C = 3.2 pg), *B*. *asparagoides* (4C = 2.3 pg) and *B. clavata* (4C = 2.2 pg).

## Discussion

### Nuclear DNA content estimates

Sister taxa such as *B*. *clavata*-*B*. *asparagoides* and *A*. *armata*-*A*. *taxiformis* [6] have similar DNA values (Table 2). *B*. *clavata* and *B*. *asparagoides* present the lowest 2C mean values of all the species studied (2C = 1.2 pg), while *A*. *armata* and *A*. *taxiformis* present the highest values (2C = 1.6/1.7 pg). Regarding *B. hamifera*, the 2C mean value (2C = 1.4 pg) is between the two groups. These results agree with the unclear taxonomic position of this species, previously considered within the *Asparagopsis* genus [6]. The only data on DNA nuclear content of Bonnemaisoniales published in picograms corresponds to *B. hamifera* with values of 2C = 1.3 pg [28], coinciding with our results. The DNA range observed in the gametophytes (1C–4C) agrees with the range indicated by Salvador *et al.* [6] for *B. asparagoides* and *B. clavata*. However, for the sporophytes or the prostrate phases, the same authors give a range of 2C to 8C.

On the other hand, keeping in mind the 1C value of the spermatia, it can be affirmed that the nuclear genome size of *B. clavata* from Mediterranean (Girona) and Atlantic (A Coruña) coasts did not show any differences.

The presence of 4C nuclei in the Bonnemaisoniaceae gametophytes suggests the possible start of an endopolyploidy process both in vegetative cells and in carpospores. These results agree with those of Salvador *et al*. [6] in *B. asparagoides* and *B. clavata* that showed a high endopolyploidy level in the axial cells of the gametophytes, as well as in the carpospores. These authors give DNA values for the carpospores of up to 32C in *B. asparagoides* and up to 16C in *B. clavata*.

### Molecular phylogeny and patterns of genome size variation

**Figure 1:**
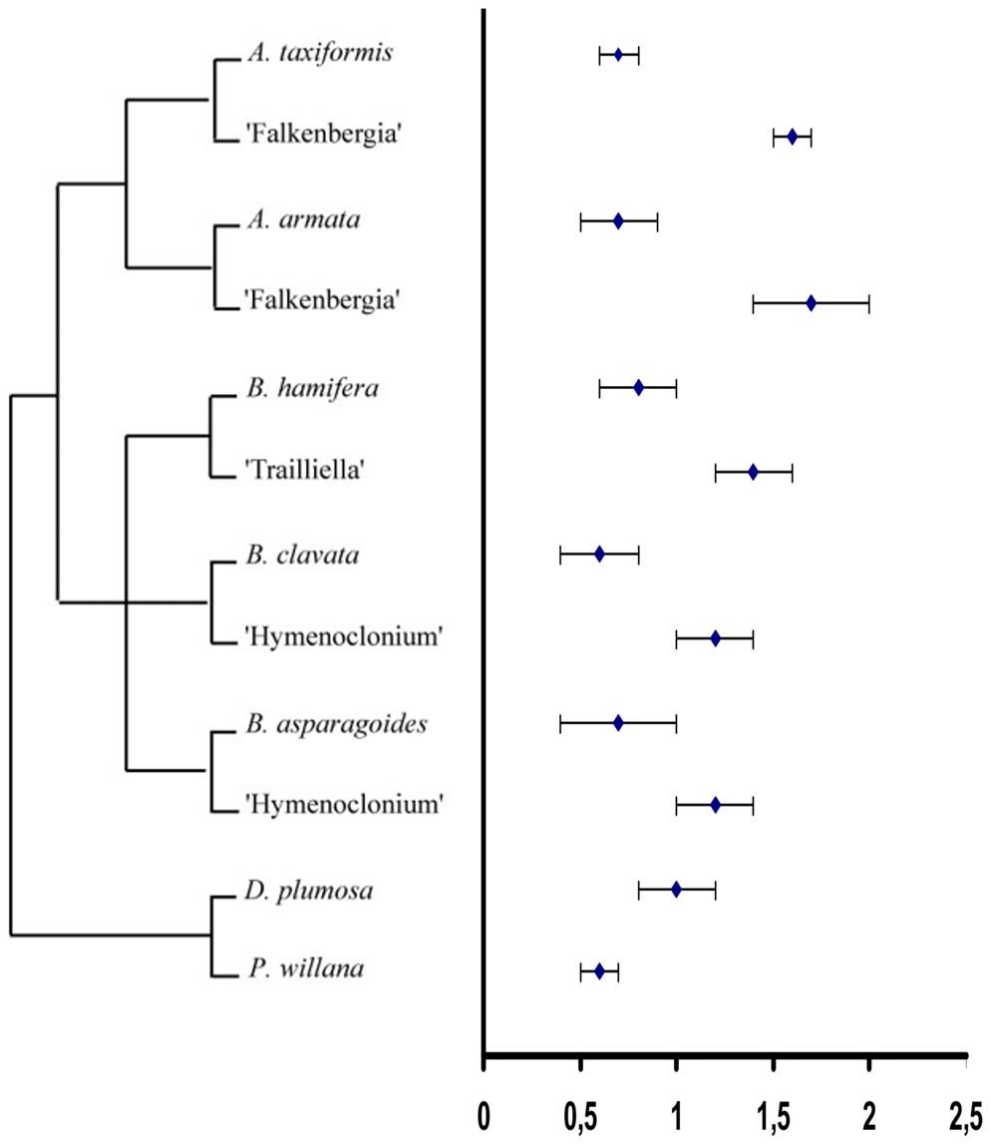
Nuclear DNA contents in picograms (pg) superimposed on a consensus molecular phylogenetic tree for Bonnemaisoniales on the basis of supported clades in published phylogenies [20], [8], [21], [6]. (○) 1C nuclear DNA contents. (•) 2C nuclear DNA contents.

A phylogenetic hypothesis for Bonnemaisoniales [20], [8], [21], [6] provides a picture of nuclear genome size evolution among these taxa (Fig. 1). Southern hemisphere genera *Delisea* and *Ptilonia* are a sister group to a *Bonnemaisonia-Asparagopsis* clade according to the phylogenetic tree resulting from SSU analysis [8]. Results of the present study indicate 1C nuclear genome sizes in Bonnemaisoniaceae (0.5–0.8 pg) within similar range observed in other members of the Florideophycidae [28].

*Asparagopsis armata* and *A*. *taxiformis* have become widely distributed in Europe as an alien introduction [7], [49], [9] and fit the definition of a marine invader [50]. The highest 2C levels observed in their respective tetrasporophytes could be related with the fact that in *Asparagopsis* this phase (“Falkenbergia” type) is the most resilient [10] and the primary means of dispersal [8].

Karyological studies limited to three species, *Asparagopsis armata* (n = ca. 20), *Bonnemaisonia asparagoides* (n = ca. 18, n = ca. 20, n = ca. 30) and *Bonnemaisonia hamifera* (n = 20–25) [51], show that the *n* chromosome number is variable. Reported chromosome complements of *n* = 10 in *A. armata* [52] should be reinvestigated. In red algae, the hypothesised basal (ancestral) nucleotype is characterized both by small genome sizes and small chromosome complements [28]. Chromosome complements greater than *n* = 10 probably reflect ancestral polyploidy events [51], [29]. Due to the variation of the chromosome number and nuclear DNA content estimates in the present study, we can suggest one or more instances of aneuploidy following an ancestral polyploidy event [53], [54], [55] as a possibility.

### Nuclear DNA content variation associated with a diplobiontic life history

Considerable life history variations have been reported in species of Bonnemaisoniales [22], [4], [56]. Culture studies suggest intraspecific variability in the development and life history of *Delisea pulchra* (Greville) Montagne [57]. In *Atractophora* and *Naccaria*, gametophytes develop directly from the prostrate protonemal stage produced from carpospores [25]. *Asparagopsis taxiformis* and *A. armata* have an alternation of generations with a ‘Falkenbergia’ tetrasporophyte [1], [2], [5]. *Bonnemaisonia hamifera* alternates with a ‘Trailliella’ tetrasporophyte [26] and *Bonnemaisonia geniculata* Gardner is reported to have a different type of tetrasporophyte [3]. In contrast, recent research of *B. asparagoides* and *B. clavata* confirms direct development of gametophytes from the prostrate ‘Hymenoclonium’ phase following vegetative meiosis [6].

The DNA-localizing fluorochrome DAPI and microspectrophotometry have been used to demonstrate variations in nuclear DNA levels consistent with an alternation of haploid and diploid phases in red algae associated with a sexual life cycle [35], [40], [29]. In the present study, no evidence of tetrasporogenesis was observed in either collected or cultured material. However, in comparing the mean values obtained between phases, the gametophytes showed a 1C range of 0.6–0.8 pg whereas their prostrate/sporophytic phases (‘Falkenbergia’, ‘Hymenoclonium’ and ‘Trailliella’) had a 2C range of 1.2–1.7 pg (Table 2). In addition, the 1C values observed in the gametophytes are corroborated by the 1C value observed in the spermatia (0.5–0.7 pg). These results confirm the alternation of haploid and diploid phases suggested by culture studies [2], [23], [26], [4], [6], but not clearly demonstrated by previous cytological research [58], [59], [4] in the Bonnemaisoniales.

Therefore, in addition to contributing the nuclear DNA content values of 7 Bonnemaisoniaceae species, this study confirms the alternation of nuclear phases and reports significant information about the understanding of the life histories of this group, where several variations have been described.
